# „Stein‑, Bein- und Magenpein“: Atypische Ursache einer Hyperkalziämie

**DOI:** 10.1007/s11560-022-00586-7

**Published:** 2022-06-23

**Authors:** M. Ganz, C. Gross, F. Gehringer, T. Wiech, A. Ambach, P. R. Mertens, J. Schiefer

**Affiliations:** 1 Universitätsklinik für Nieren- und Hochdruckkrankheiten, Diabetologie und Endokrinologie, Otto-von-Guericke-Universität Magdeburg, Leipziger Str. 44, 39120 Magdeburg, Deutschland; 2 Institut für Pathologie, Universitätsklinikum Hamburg-Eppendorf, Hamburg, Deutschland; 3 Universitätshautklinik, Otto-von-Guericke-Universität Magdeburg, Magdeburg, Deutschland

## Anamnese

Die Vorstellung des 49-jährigen Patienten erfolgte in unserer Notaufnahme nach Zuweisung durch den hinzugezogenen niedergelassenen Nephrologen bei schwerer Hyperkalziämie über 3 mmol/l (Referenz: 2,15–2,5 mmol/l) und stark eingeschränkter Nierenfunktion mit einer glomerulären Filtrationsrate (GFR) von 21,5 ml/min (Referenz: ≥ 90 ml/min/1,73 m^2^). Zuvor stellte sich der Patient beim Hausarzt bei Müdigkeit, Abgeschlagenheit, Gewichtsverlust 16 kg in 6 Monaten sowie Belastungsdyspnoe NYHA(New York Heart Association)-Grad 2 vor. Diese Symptomatik wurde zuvor im Rahmen eines Long-COVID(„coronavirus disease“)-Syndroms bei durchgemachter SARS-CoV-2(„severe acute respiratory syndrome coronavirus 2“)-Infektion im Dezember 2020 interpretiert. Weitere Vorerkrankungen des Patienten umfassen eine langjährige arterielle Hypertonie sowie eine Hemiparese links bei ischämischem Hirninsult im Jahr 2011.

## Klinischer Befund

**Figure 1:**
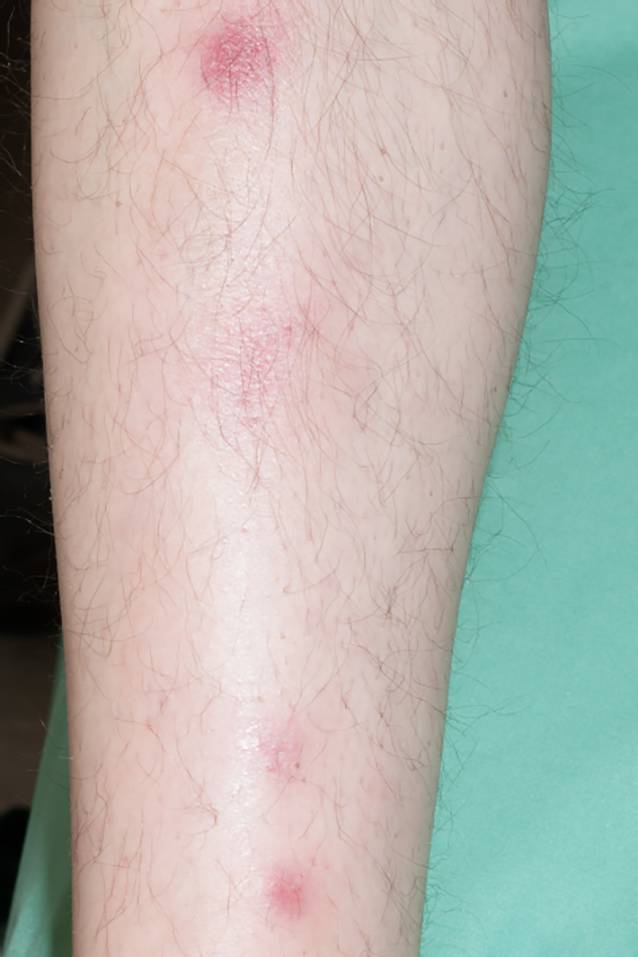

Reduzierter Allgemeinzustand, 182 cm, 70 kg, Blutdruck: 120/80 mm Hg, Puls: 94/min, Lunge bds. mit vesikulärem Atemgeräusch, keine Nebengeräusche. Herztöne rein, rhythmisch, keine Herzgeräusche. Weiche livide rot-violette Knötchen am linken Unterarm und prätibial (Abb. 1). Schilddrüse, Leber und Milz nicht vergrößert tastbar. Klinisch Exsikkose. Kraftgrad bei bekannter Hemiparese: 3/5 (links) bzw. 5/5 (rechts).

## Aufnahmelabor

Serumgesamtkalzium: 3,49 mmol/l (2,15–2,5 mmol/l), ionisiertes Kalzium: 1,91 mmol/l (1,15–1,29 mmol/l). Normwertiges Serumphosphat (1,19 mmol/l [0,81–1,45 mmol/l]). Geschätzte glomeruläre Filtrationsrate (eGFR): 30,9 ml/min/1,73 m^2^ Körperoberfläche (KOF; CKD-EPI[Chronic Kidney Disease Epidemiology Collaboration]-Formel), Harnstoff: 7,2 mmol/l (Referenz: ≤ 7,3 mmol/l). Mikroalbuminurie mit einer Albumin-Kreatinin-Ratio von 70 mg/mmol Kreatinin (Referenz: < 2,5 mg/mmol), normale Urin-Gesamtprotein-Kreatinin-Ratio (12,7 mg/mmol Kreatinin [Referenz: < 30 mg/mmol]). Immunfixation im Serum ohne Nachweis eines monoklonalen Proteins.

### Blutbild

Hämoglobin: 7,2 mmol/l (Referenz: 8,4–10,9 mmol/l), Leukozyten: 3,71 Gigapartikel (Gpt)/l (Referenz: 3,7–9,8 Gpt/l), Thrombozyten: 216 Gpt/l (Referenz: 146–328 Gpt/l). Segmentkernige Neutrophile mit 77,2 % relativ erhöht (Referenz: 40–75 %), Lymphozyten mit 6,70 % relativ erniedrigt (Referenz: 17–47 %). C‑reaktives Protein mit 64 mg/l erhöht (Referenz: < 5 mg/l).

## Weitere Diagnostik

Zur weiteren Abklärung hinsichtlich der Hyperkalziämie wurden ACE („angiotensin-converting enzyme“), der lösliche („soluble“) Interleukin-2-Rezeptor (sIL2R) sowie Calcidiol und Calcitriol im Blut bestimmt. ACE, sIL2R und Calcitriol zeigten sich erhöht, Calcidiol erniedrigt. Aufgrund der typischen Laborkonstellation und bei supprimiertem Parathormon (PTH) ergab sich der Verdacht auf eine granulomatöse Grunderkrankung.

**Verdachtsdiagnose:** Hyperkalziämie bei granulomatöser Grunderkrankung (Sarkoidose, Morbus Boeck)

**Figure 2:**
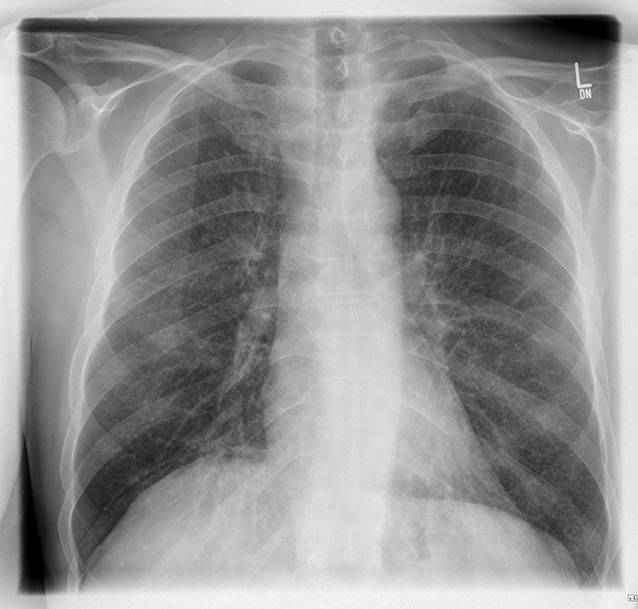

Es wurde eine röntgenologische Übersichtsaufnahme des Thorax angefertigt (Abb. 2), welche einen unauffälligen Befund ergab. Die kapilläre Blutgasanalyse zeigte bei Raumluft eine Normoxämie und Normokapnie bei normaler Atemfrequenz. In der Bodyplethysmographie mit Messung der Diffusionskapazität konnten eine relevante Obstruktion, Restriktion und Diffusionsstörung der Lunge ausgeschlossen werden. In Zusammenschau der Befunde erfolgte bei akuter Nierenschädigung und noch ungeklärter Genese der Hyperkalziämie eine sonographiegestützte Nierenbiopsie.

## Histologie Nierenbioptat

**Figure 3:**
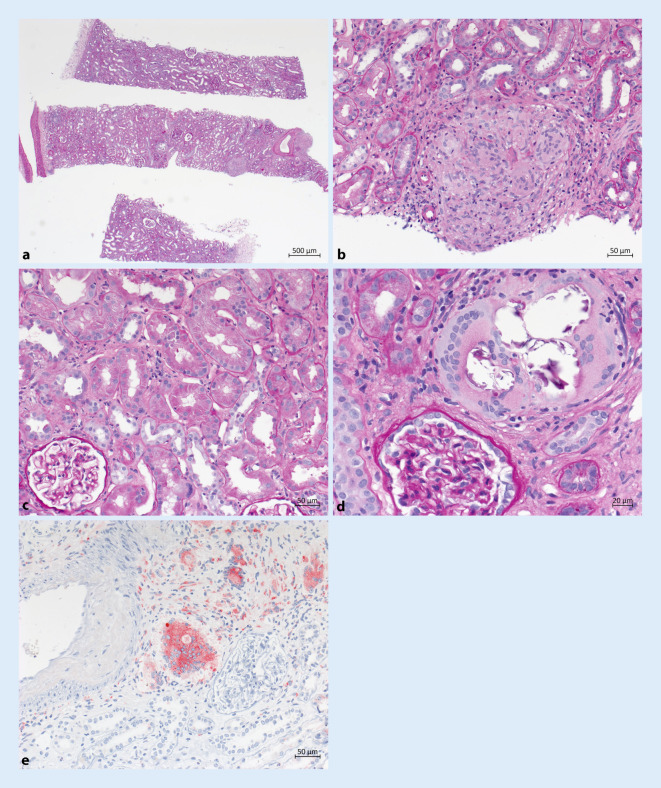

Fokale epitheloidzellig-granulomatöse interstitielle Nephritis, Nephrokalzinose, kein Nachweis einer primären glomerulären Erkrankung (Abb. 3).

## Wie lautet Ihre Diagnose?

Vor dem Hintergrund der gleichzeitig bestehenden systemischen Hyperkalziämie wurde die Diagnose einer Sarkoidose mit renaler Manifestation gestellt. An der Haut präsentiert sich ein Erythema nodosum im Rahmen der Grunderkrankung.

In der Histologie der Nierenbiopsie zeigte sich ein typisches Muster mit

- interstitieller lymphoider Infiltration und variablem Ausmaß interstitieller Fibrose und tubulärer Atrophie;
- bevorzugt peritubulären nichtverkäsenden Granulomen mit Nachweis von Makrophagen, mehrkernigen Riesenzellen sowie zentral prädominant CD4-positiven Lymphozyten;
- dystoper Kalzifikation (peritubulär, interstitiell);
- negativer Untersuchung hinsichtlich säurefester Stäbchen.

Typisch für die Sarkoidose ist die primär intrathorakale Manifestation (Lunge, mediastinale Lymphknoten), jedoch sind weitere heterogene Organbeteiligungen – durchaus mit systemischer Wirkung – möglich (Tab. 1).

**Table 1:** 

Organsystem	Prozentuale Verteilung
Lunge	95
Haut	15,9
Lymphknoten	15,2
Augen	11,8
Leber	11,5
Milz	6,7
Nervensystem	4,6
Speicheldrüsen	3,9
Knochenmark	3,9
Hyperkalziämie	3,7
HNO	3,0
Herz	2,3
Niere	0,7
Knochen/Gelenke	0,5
Muskulatur	0,4

*HNO* Hals-Nasen-Ohren

Bei unauffälligem Befund des Röntgenthorax, normalen kapillären Blutgasen, normwertiger Lungenfunktionsuntersuchung sowie wegeweisender Histologie der Nierenbiopsie wurde auf eine Bronchoskopie zunächst verzichtet.

## Therapie und Verlauf

Es wurde eine orale Prednisolontherapie mit 1 mg Prednisolon pro kg Körpergewicht pro Tag ordiniert. Es erfolgte eine multimodale konservative kalziumsenkende Therapie. Die Hyperkalziämie war schnell rückläufig, die Nierenfunktion des Patienten besserte sich nach Therapieeinleitung.

In der ambulanten Nachkontrolle 1 Monat später war die Nierenfunktion nur noch leichtgradig eingeschränkt (GFR: 78,0 ml/min, Stadium G2 nach KDIGO [Kidney Disease: Improving Global Outcomes]). Im weiteren Verlauf erfolgte eine schrittweise Reduktion der Prednisolondosis.

## Diskussion

Die Sarkoidose ist eine Multisystemerkrankung, deren Diagnose sich auf typische röntgenologische, laborchemische und histologische Befunde sowie nicht zuletzt auf den Ausschluss anderer Krankheitsbilder stützt. Die Ätiologie und die Pathogenese sind noch nicht vollständig geklärt [1, 2]. Diskutiert werden als Initiator die initiale Phagozytose und Antigenpräsentation eines noch nicht definierten Antigens (Autoantigen oder fremdes Agens) vor dem Hintergrund einer gestörten Regulation hinsichtlich der Terminierung der Immunreaktion (gestörte Funktion von regulatorischen T‑Zellen) und einer verminderten Antigen-Clearance. Polymorphismen in Genen wie z. B. *BTNL2* („butyrophilin-like 2“) sowie gewisse HLA(„human leukocyte antigen“)-DR- und HLA-B-Varianten gehen mit einem erhöhten Krankheitsrisiko einher. Bei COVID-19(„coronavirus disease 2019“)-Erkrankten wurden vereinzelt histopathologisch sarkoidoseähnliche Läsionen in Lungenbioptaten gefunden. Zudem führt eine SARS-CoV-2-Infektion zu einer gestörten Autophagie, welche eine Rolle im postulierten Entstehungsmechanismus der Sarkoidose spielen könnte [3]. Inwieweit die bei unserem Patienten vorausgegangene COVID-19-Infektion mit der einige Monate später diagnostizierten Sarkoidose zusammenhängt, bleibt jedoch unklar.

Die klassische initiale pulmonale Manifestation zeigt sich bei etwa 90 % der Patienten [4]. Eine Rarität hingegen ist die renale Erstmanifestation [5].

**Diagnose:** Hyperkalziämie bei Sarkoidose mit renaler Manifestation und Erythema nodosum

Qualitativ hochwertige Evidenz zur Therapie der extrapulmonalen Sarkoidose ist fast nur hinsichtlich der kardialen Sarkoidose vorhanden [6]. Hier wird eine initial hoch dosierte Prednisolontherapie mit schrittweiser Dosisreduktion empfohlen (Therapiedauer: mindestens 24 Monate). Als Reserveoptionen stehen Azathioprin und Methotrexat zur Verfügung. Als Rescue-Therapie besteht die Option der Tumornekrosefaktor(TNF)-alpha-Inhibitoren, die jedoch mit relevanten Nebenwirkungen behaftet sein kann. Differenzialdiagnostisch müssen v. a. bei der pulmonalen Sarkoidose mykobakterielle Infektionen, Pilzinfektionen (Histoplasmose, *Pneumocystis jiroveci*) und Pneumokoniosen mit besonderem Augenmerk auf die Berylliose sowie zahlreiche weitere Erkrankungen erwogen werden [7].

## Fazit für die Praxis

- ein Post-COVID(„coronavirus disease“)-Syndrom ist unspezifisch, die Symptomatik kann durch andere schwere Erkrankungen, wie in diesem vorliegenden Fall einer granulomatösen Erkrankung, bedingt sein.
- Eine Sarkoidose lässt sich nicht allein durch pulmonale Diagnostik ausschließen, primär extrapulmonale Manifestationen sind möglich. Gezielte Untersuchungen (z. B. durch Nierenbiopsie, Kardio-MRT [Magnetresonanztomographie], nuklearmedizinische Untersuchungen) und der Ausschluss alternativer Diagnosen führen zur Diagnose einer extrapulmonalen Sarkoidose.
- Die renale Manifestation einer Sarkoidose kann bei Korrektur der Hyperkalziämie einen guten Verlauf mit Rekonstitution der Funktion und Auflösung der Nephrokalzinose nehmen.
- Eine immunsuppressive Therapie ist an den Verlauf der Sarkoidoseerkrankung anzupassen.
